# Review Department

**Published:** 1890-03

**Authors:** 


### REVIEW DEPARTMENT.

AMERICAN NATURALIST. Philadelphia: Ferris Bros.

The January, 1890, number comes to us on time, and is full of interesting articles, not only to the naturalist but to the general reader. An article by Mr. Stearn, entitled "The Effects of Musical Sounds upon Animals," is suggestive and contains some very curious accounts of the manner in which dogs and other animals are affected by musical sounds. The author has made experiments through the medium of music with a view of discovering to what extent a fellow feeling exists between man and certain animals. The results are not found in the part of the article published in this number, so that the readers of the JOURNAL, especially psychologists, may look forward to the coming number with interest. The other articles are "On Excavations Made by Sea Urchins" by Mr Fewkes; "The History of Garden Vegetables," by Mr. Sturtevant; an editorial on "Darwinism" and general notes on everything new in the natural sciences.

REMARKS UPON EXTINCT MAMMALS OF THE UUITED STATES. By R. W. SHUFELDT, 1889.

This interesting pamphlet, with a dozen plates, is well worth reading either by the student, whose other duties prevent him from taking up the wonderful work of paleontology, or by the lay reader, who will find in it, in a concise and attractive way, an outline of some of the progenitors of ourselves and the animals around us. The *Tinoceros* is pictured to make us see the banks of the Nile running through the Western prairies. A clear series of sketches of two, three, and four-toed horses accompanies an article on the "Ancestry of the Horse." Ancient whales and coryphodons, half-apes and lemurs, the great sabre-toothed tiger, hairy mammoths and sea-cows form the other subjects. Reference is made to some of the more recent animals which are becoming extinct.

